# Immunogenicity and reactogenicity of accelerated regimens of fractional intradermal COVID-19 vaccinations

**DOI:** 10.3389/fimmu.2022.1080791

**Published:** 2023-01-17

**Authors:** Suvimol Niyomnaitham, Suparat Atakulreka, Patimaporn Wongprompitak, Katherine Kradangna Copeland, Zheng Quan Toh, Paul V. Licciardi, Kanjana Srisutthisamphan, Laddawan Jansarikit, Kulkanya Chokephaibulkit

**Affiliations:** ^1^ Siriraj Institute of Clinical Research, Bangkok, Thailand; ^2^ Department of Pharmacology, Faculty of Medicine Siriraj Hospital, Mahidol University, Bangkok, Thailand; ^3^ Department of Immunology, Faculty of Medicine Siriraj Hospital, Mahidol University, Bangkok, Thailand; ^4^ Department of Biological Sciences, Faculty of Science, Mahidol University International College, Nakhon Pathom, Thailand; ^5^ Infection and Immunology, Murdoch Children’s Research Institute, Parkville, VIC, Australia; ^6^ Department of Pediatrics, The University of Melbourne, Parkville, VIC, Australia; ^7^ National Center for Genetic Engineering and Biotechnology (BIOTEC), National Science Development Agency (NSTDA), Pathumthani, Thailand; ^8^ Department of Pediatrics, Faculty of Medicine Siriraj Hospital, Mahidol University, Bangkok, Thailand

**Keywords:** fractional dose, intradermal, accelerated regimen, COVID-19 vaccination, immunogenicity, heterologous regimen, Thailand

## Abstract

**Introduction:**

This phase I study explored the immunogenicity and reactogenicity of accelerated, Q7 fractional, intradermal vaccination regimens for COVID-19.

**Methods:**

Participants (n = 60) aged 18-60 years, naïve to SARS-CoV-2 infection or vaccination, were randomly allocated into one of four homologous or heterologous accelerated two-dose, two-injection intradermal regimens seven days apart:(1) BNT162b2-BNT162b2(n= 20),(2) ChAdOx1- BNT162b2 (n = 20), (3) CoronaVac-ChAdOx1 (n = 10), and (4) ChAdOx1-ChAdOx1 (n = 10). CoronaVac and ChAdOx1 were 20%, and BNT162b2 17%, of their standard intramuscular doses (0.1 mL and 0.05 mL per injection, respectively). Humoral immune responses were measured through IgG response towards receptor binding domains (RBD-IgG) of ancestral SARS-CoV-2 spike protein and pseudovirus neutralization tests (PVNT50). Cellular immune responses were measured using ELISpot for ancestral protein pools.

**Results:**

Immunogenicity was highest in regimen (2), followed by (1), (4), and (3) 2 weeks after the second dose (P < 0.001 for anti-RBD-IgG and P= 0.01 for PVNT50). Each group had significantly lower anti-RBD IgG (by factors of 5.4, 3.6, 11.6, and 2.0 for regimens (1) to (4), respectively) compared to their respective standard intramuscular regimens (P < 0.001 for each). Seroconversion rates for PVNT50 against the ancestral strain were 75%, 90%, 57% and 37% for regimens (1) to (4), respectively. All participants elicited ELISpot response to S-protein after vaccination. Adverse events were reportedly mild or moderate across cohorts.

**Discussion:**

We concluded that accelerated, fractional, heterologous or homologous intradermal vaccination regimens of BNT162b2 and ChAdOx1 were well tolerated, provided rapid immune priming against SARS-CoV-2, and may prove useful for containing future outbreaks.

## 1 Introduction

Intradermal (ID) injection, or the administration of drugs into the dermis, is an alternative method of vaccination to conventional intramuscular (IM) or subcutaneous (SC) routes (1, 2). The dermis and epidermis are rich in antigen-presenting cells (or APCs, *i.e.*, dermal dendritic cells [DDCs]), that dramatically enhance innate and adaptative immune responses (1, 3–7). Fractional doses 10-20% of their IM or SC dosages are generally used for ID administration (5–8). Such dose reductions could help increase vaccine supply and availability as well as reduce vaccination costs by 60-80% compared to IM regimens (3, 8, 9). Addressing these factors are crucial to navigating global vaccine shortages, particularly in low- and middle-income countries (3).

Fractional dosing also reduces reactogenicity and systemic adverse events (AEs) due to dose-dependency (2, 3). AEs are reportedly milder and more transient for ID administration, with local reactions being more common compared to conventional IM or SC routes, rendering it a safer option (3, 5).

Accelerated, dose-sparing approaches towards mass immunization broadens vaccine coverage and population immunity, preventing the spread of SARS-CoV-2 (2, 5). Over the last couple decades, more than 90 clinical trials for 11 different diseases (*e.g.*, Rabies, Poliovirus, Yellow Fever, Hepatitis A and B, seasonal Influenza, *etc.*) were explored to establish whether accelerated schedules for ID regimens could induce sufficient immunity compared to IM regimens (2, 3, 7, 10). Many studies found similar or enhanced immunogenicity in ID regimens compared to conventional IM or SC routes (2, 5, 10). Accelerated schedules generated rapid immunity that facilitated disease prophylaxis post-exposure, hence routine recommendations for rabies (11).

CoronaVac and ChAdOx1-nCov19 were introduced into Thailand early on during the pandemic; BNT162b2 quickly followed thereafter. This study explored the immunogenicity and reactogenicity of accelerated, fractional, ID dosing regimens of homologous and heterologous COVID-19 vaccines within a Thai population.

## 2 Material and methods

### 2.1 Study design and participants

This single-center, randomized, prospective, open-labelled, pilot cohort study enrolled healthy adults aged 18-60 years during September to December of 2021. Participants naïve for SARS-CoV-2 infection, capable of adhering to scheduled fractional dosing regimens, with an ability to understand Thai, and to self-report digitally were included in the study. Exclusion criteria included those that: were previously infected with SARS-CoV-2; received prophylactic or investigational COVID-19 treatment; received blood, plasma, immunoglobulins, or antibodies within 90 days of the study; had a history of severe drug or vaccine allergies, pre-existing comorbidities, underlying diseases, drug, or substance abuse; were pregnant; and were immunocompromised or receiving immunosuppressive agents. These study protocols were approved by the Human Research Protection Unit, Faculty of Medicine Siriraj Hospital, Mahidol University (COA: MU-MOU 704/2021) and registered under Thailand’s Clinical Trial Registry (registration number: TCTR20210904004, https://www.thaiclinicaltrials.org/). All participants provided written informed consent.

### 2.2 Study procedure

Initially, 10 participants were randomly assigned into 4 groups each to receive two homologous or heterologous ID doses of CoronaVac (Sinovac), ChAdOx1 nCoV-19 (AstraZeneca), or BNT162b2 (Pfizer-BioNTech) 7 days apart. The vaccine regimens (first-second dose) for each arm were: BNT162b2-BNT162b2 (Group 1), ChAdOx1-BNT162b2 (Group 2), CoronaVac-ChAdOx1 (Group 3), and ChAdOx1-ChAdOx1 (Group 4). Participants received two injections of each vaccination, one in each arm within the deltoid region. The dosage for each injection was 10-20% of conventional IM doses based on previously published literature (3). This entailed 0.1 mL for CoronaVac and ChAdOx1 (20% of standard 0.5 mL IM dosage) and 0.05 mL for BNT162b2 (17% of standard 0.3 mL IM dosage). Blood samples were collected before each dose and 2 weeks after the second dose for immunologic evaluations, including: anti-receptor binding domain of Wuhan strain S1-subunit spike IgG (anti-RBD IgG), neutralizing antibodies (NAb) for Wuhan, and cellular immune responses against Wuhan. As an exploratory outcome, NAb levels were also measured against omicron subvariants. Baseline samples were also tested for SARS-CoV-2 anti-nucleocapsid protein (anti-NP) antibodies to determine prior natural infection. Participants across all four regimens were instructed to digitally self-assess and report their solicited local and/or systemic AEs after each dose. Solicited local AEs entailed injection site reactions, while systemic AEs included: myalgia, fatigue, headache, fever, diarrhea, and nausea. Both types of AEs were graded numerically on scales of 1 to 4 based on the Common Terminology Criteria for Adverse Events (Version 5.0) by the United States National Cancer Institute (NCI/NIH). Mild scores (1) did not interfere with patients’ activities, moderate scores (2) interfered with patients’ activity, severe scores (3) prevented daily activities, and emergency scores (4) required hospitalization 4 (12).

Preliminary analyses of anti-RBD IgG were performed for all regimens and participants. Evidence of positive anti-NP or anti-RBG IgG at baseline were excluded. In the extended phase, additional participants were recruited to the two groups (10 per regimen) with the highest anti-RBD IgG measured 2 weeks after the second vaccination to meet statistical power. Additional blood sample were collected 12 weeks after the second dose for these two groups to evaluate the persistence of anti-RBG IgG.

### 2.3 Chemiluminescent microparticle assay for anti-SARS-CoV-2 RBD IgG and anti-NP of ancestral strain

Plasma samples were isolated using sodium citrate and stored at -80°C. A chemiluminescent microparticle assay (CMIA) was used to determine anti-RBD and anti-NP through SARS-CoV-2 IgG II Quant (Abbott Laboratory System, Illinois, US) on the ARCHITECT i System. Antibody levels were linearly measured between 21.0-40,000.0 arbitrary units per mL (AU/mL), and subsequently converted to binding antibody units per mL (BAU/mL) per WHO’s International Standards and an equation provided by the manufacturer (BAU/mL = 0.142 × AU/mL). Seropositivity was defined by cutoff values ≥ 50 AU/mL (7.1 BAU/mL). Antibody response against SARS-CoV-2 NP protein was determined using baseline plasma samples through CMIA SARS-CoV-2 IgG (Abbott, List No. 06R86) on the ARCHITECT i System.

### 2.4 Pseudovirus neutralization test for ancestral Wuhan strain and omicron subvariants

PVNT was carried out as described previously at the National Center of Genetic Engineering and Biotechnology, Thailand (13). Assays were performed against the ancestral Wuhan strain, and omicron subvariants BA.1, BA.2, and BA.5. Antibody titers (PVNT_50_) were calculated using GraphPad Prism 9 version 9.2.0 (GraphPad Software, CA, USA) to interpolate the point at which pseudovirus infectivity had been reduced by 50% of the value found for no serum control samples. The limit of detection (LOD) was 1:40.

### 2.5 Cell-mediated immune response by ELISpot assay to ancestral Wuhan strain

T-cell responses were assessed using human interferon-gamma (IFN-γ) ELISpot kits according to the manufacturers’ instruction (Mabtech AB, Nacka Strand, Sweden – as previously described (14)). This entailed the use of two peptide pools : (1) an S-defined peptide pool (Mabtech) consisting of 100 peptides from spike (S) protein (purity of 90%); and (2) an NMO-defined peptide pool (Mabtech) consisting of 101 peptides from nucleocapsid (N), membrane (M), open reading frame (ORF) 1, non-structural protein (nsp) 3, ORF-3a, ORF-7a, and ORF-8 proteins (purity of 87%). The ELISpot plates were read using IRIS (Mabtech) and spots were analyzed using Apex software 1.1 (Mabtech) and converted to spot-forming units (SFU) per million cells. The cut-off for positive response was set as 20 SFU.

### 2.6 Statistical analysis

Participants positive for anti-NP and anti-RBD at baseline were excluded from the analysis. Immunological endpoints (anti-SARS-CoV-2 RBD IgG, PVNT_50_, and ELISpot SFU) were reported as geometric means (GMs), with 95% confidence intervals (CI). PVNT_50_ titers that were lower than LOD of 40 were assigned a value of 20. As all participants were seronegative at baseline, seroconversion was determined as becoming seropositive (anti-RBD IgG ≥ 50 AU/mL, PVNT_50_ ≥ 40) two weeks after the second vaccination. Sample sizes of 20 per regimen would provide the ability to detect differences between ID and conventional IM regimens with 85% confidence. GraphPad Prism 9 version 9.2.0 (GraphPad Software, CA, USA) was used to perform unpaired *t*-tests for GM comparisons between treatment regimens. ANOVA and STATA version 17 (StataCorp, LP, College Station, TX, USA) were used to examine GMs for different regimens. Endpoints of AEs were presented as frequencies and Chi-square tests were used to test for statistical differences between endpoints. We represented this information as GMs of anti-RBD-IgG and AE frequency, similar to another report of ours regarding IM regimens in healthy volunteers (15).

## 3 Results

**Figure 1:**
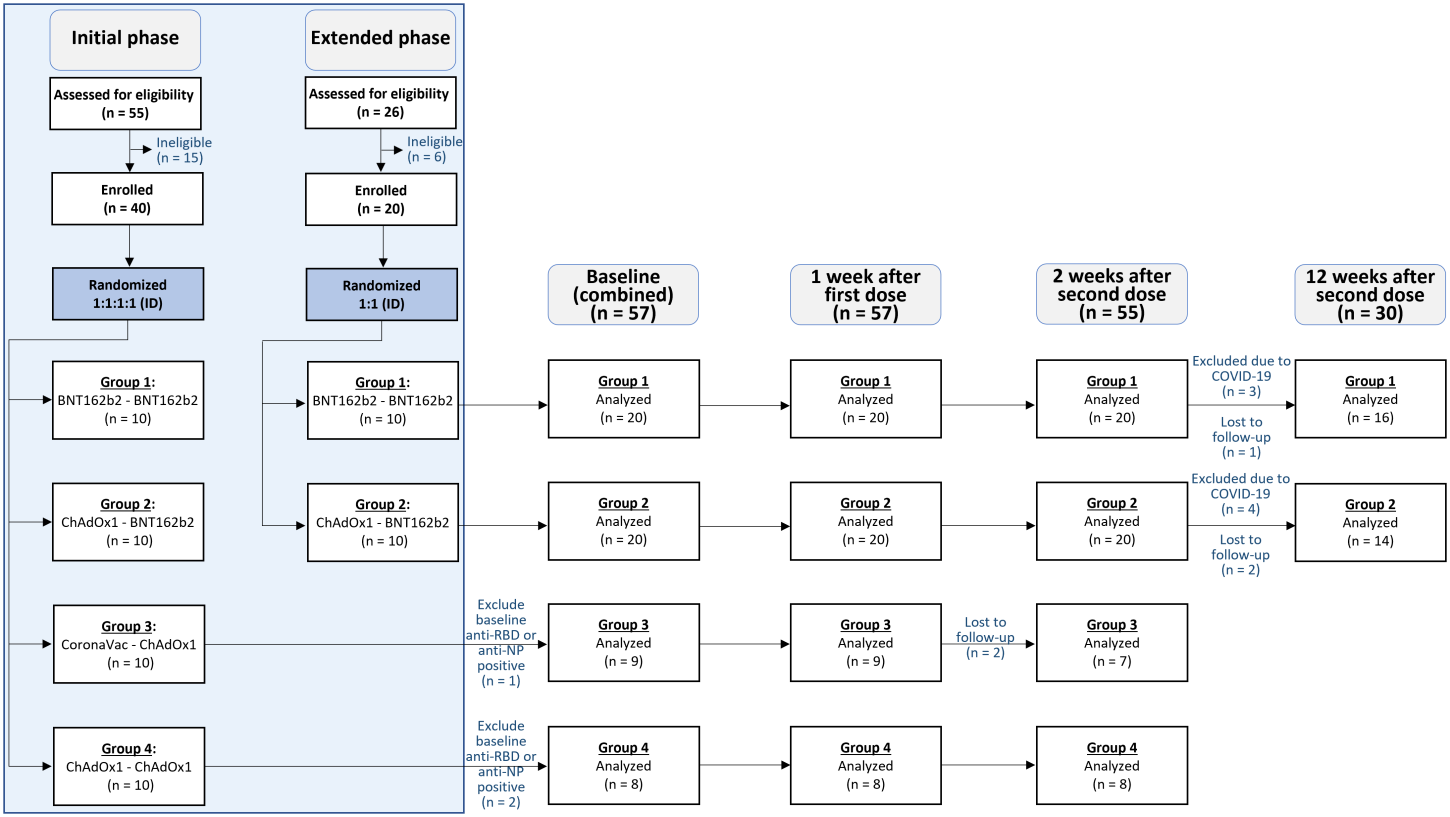
Consort diagram. 55 participants were assessed for eligibility in the initial phase, and 40 were included and randomized (1:1:1:1) to receive accelerated intradermal regimens in the study. An additional 26 participants were assessed for eligibility in the extended phase, and 20 were included and randomized (1:1) into two of the four accelerated intradermal vaccine regimens. Participants were assessed thereafter at baseline (n = 57), 1 week after the first dose (n = 57), 2 weeks after the second dose (n = 55), and 12 weeks after the second dose (n =30).

55 participants were screened during the initial phase of the study, and 40 were enrolled. Following the preliminary analysis of this initial phase, Groups 1 and 2 induced the highest immunogenic responses (highest anti-RBD IgG). An additional 26 participants were screened, and 20 subsequently enrolled in these two groups for the extended phase of this study. This amounted to a total of 60 participants enrolled in the study, with 3 excluded from its analysis due to positive baseline anti-NP or anti-RBD IgG antibody values (see **Figure 1**). Among the 57 participants included in the analysis, 38 (67.7%) were male, with a median BMI of 23.1 (IQR of 21.2-25.9), and median age of 35 (interquartile range [IQR] of 28-45) years. No significant differences in demographics were observed across groups (see **Table 1**).

**Table 1:** Baseline characteristics of the participants receiving accelerated regimens of intradermal COVID-19 vaccinations.

		Types of vaccines					
First dose (ID) - Second dose (ID)		Total	BNT162b2 - BNT162b2	ChAdOx1 - BNT162b2	CoronaVac - ChAdOx1	ChAdOx1 - ChAdOx1	*p*-value
Number of participants	n %	57 (100.00)	20 (35.09)	20 (35.09)	9 (15.79)	8 (14.03)	
Age (years)	Median (IQR)	35.00 (28.00, 45.00)	33.50 (29.00, 47.00)	34.50 (25.50, 39.00)	31.00 (28.00, 49.00)	39.50 (29.00, 48.00)	0.674
Male	n %	38 (66.67)	16 (80.00)	14 (70.00)	5 (55.56)	3 (37.50)	0.154
Body mass index (kg/m^2^)	Median (IQR)	23.10 (21.20, 25.90)	22.15 (20.15, 25.50)	23.50 (22.05, 26.85)	21.60 (20.90, 22.10)	24.35 (22.95, 27.05)	0.262

Chi-square and Kruskal-Wallis tests were used to determine p-value among those who received any of the four vaccination regimens. ID, Intradermal.

### 3.1 Anti-SARS-CoV-2 RBD IgG response to ancestral Wuhan strain

**Figure 2:**
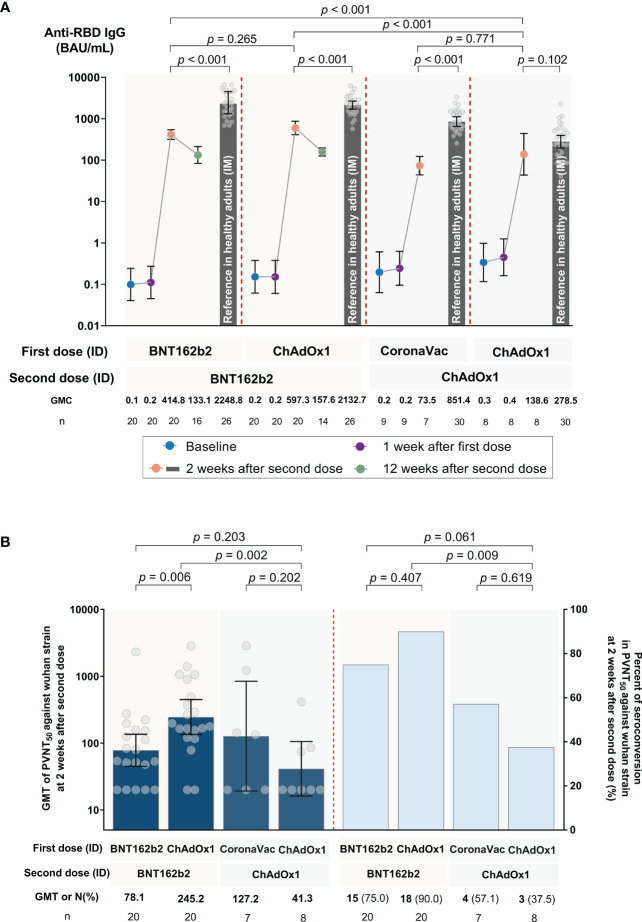
SARS-CoV-2 humoral immune responses against the ancestral Wuhan strain following accelerated regimens of fractional, ID administration. **(A)** SARS-CoV-2 RBD IgG at baseline, 1 week after the first dose, and 2 weeks after the second dose for Groups 1 to 4, as well as 12 weeks after the second dose for Groups 1 and 2. The reference bars represent the results for conventional intramuscular (IM) routes of each respective COVID-19 vaccine regimen, as obtained from a previous study (15). **(B)** Pseudovirus neutralization tests (PVNT_50_, shown in dark blue) and percent seroconversion (shown in blue bars) 2 weeks after the second dose. Error bars represent geometric means (GMs) and 95% confidence intervals (CI).

None of the participants were seropositive for anti-RBD IgG 7 days after the first dose. All were seropositive after the second dose. Anti-SARS-CoV-2 RBD IgG geometric mean concentrations (GMCs) 2 weeks after the second dose had significantly increased (*P* < 0.001) compared to baseline values for all four regimens: 414.84 (95% confidence interval (CI): 316.96, 542.96) BAU/mL for Group 1, 597.29 (95% CI: 411.37, 867.25) BAU/mL for Group 2, 73.51 (95% CI: 44.09, 122.57) BAU/mL for Group 3, and 138.56 (95% CI: 43.59-440.46) BAU/mL for Group 4 (see **Figure 2A** and **Supplementary Table 1**). No statistical significance in anti-RBD IgG was observed 2 weeks after the second vaccination as seen between Groups 1 and 2, as well as Groups 3 and 4. However, Groups 1 and 2 induced significantly higher anti-RBD IgG than Groups 3 and 4 (*P* < 0.001 for each comparison). 2 weeks after second dose, each group had significantly lower anti-RBD IgG (5.4, 3.6, 11.6, and 2.0 times lower for Groups 1 to 4, respectively) compared to reference anti-RBD IgG values from conventional IM regimens (*P* < 0.001 for each comparison, except Group 4). Anti-RBD IgG for Groups 1 and 2 decreased by 3.1 and 3.8 times, respectively, 12 weeks following the second dose.

### 3.2 PVNT_50_ antibody response against SARS-CoV-2 variants

The PVNT_50_ geometric mean titer (GMT) and seroconversion against the ancestral Wuhan strain 2 weeks after the second vaccination are shown in **Figure 2B** and **Supplementary Table 1**. Seroconversion rates varied between regimens. Higher seroconversion rates (75% and 90% for Groups 1 and 2, respectively) were observed in those vaccinated with at least one BNT162b2 dose compared to those with CoronaVac-ChAdOx1 or two-doses of ChAdOx1 (57.1% and 37.5% for Groups 3 and 4, respectively, as shown in **Figure 2B**). The exploratory outcome for the seroconversion rate and PVNT_50_ titers against omicron subvariants after two doses was low or undetectable (**Supplementary Figures 1A, B**, and **Supplementary Table 1**). These low PVNT_50_ responses against omicron were like those generated in their respective IM vaccine regimens.

### 3.3 Cell-mediated immune response by ELISpot to ancestral Wuhan strain

**Figure 3:**
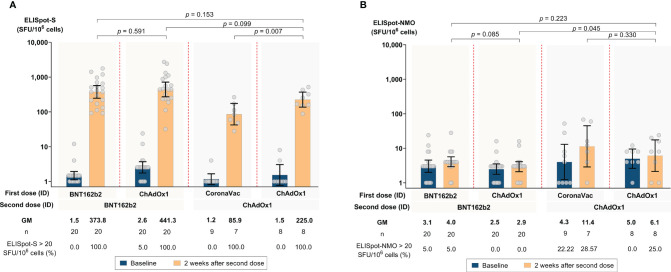
SARS-CoV-2 antigen-specific T-cell responses by ELISpot at baseline and 2 weeks after the second dose following accelerated regimens of fractional, ID administration. **(A)** Spike **(S)** protein pool-specific T-cell responses. **(B)** Nucleocapsid-membrane-open (NMO) reading frame protein pool- specific T-cell responses. Error bars represent geometric mean concentrations (GMCs) and 95% confidence intervals (CI).

All participants, except one, had negative responses at baseline. One participant had a low ELISpot response to S-protein (24 SFU). 2 weeks after the second dose, all participants across the four regimens had significant increases in IFN-γ response against S-protein. The highest GM SFU for S-protein 2 weeks after the second dose was observed in Group 2 (441.34; 95% CI 271.10, 718.47), followed by Group 1 (373.80; 95% CI 246.12, 567.72), Group 4 (224.99; 95% CI 136.63, 370.50), and Group 3 (85.88; 95% CI 42.22, 174.70) (see **Figure 3** and **Supplementary Table 1**). There were no significant differences in IFN-γ responses against S-protein between Groups 1, 2, and 4. Group 3 had a significantly lower response compared to the other three groups (*P* = 0.002) (see **Supplementary Table 1**). There was no significant increase in IFN-γ response against NMO proteins for all four groups (see **Figure 3** and **Supplementary Table 1**).

### 3.4 Adverse events

**Figure 4:**
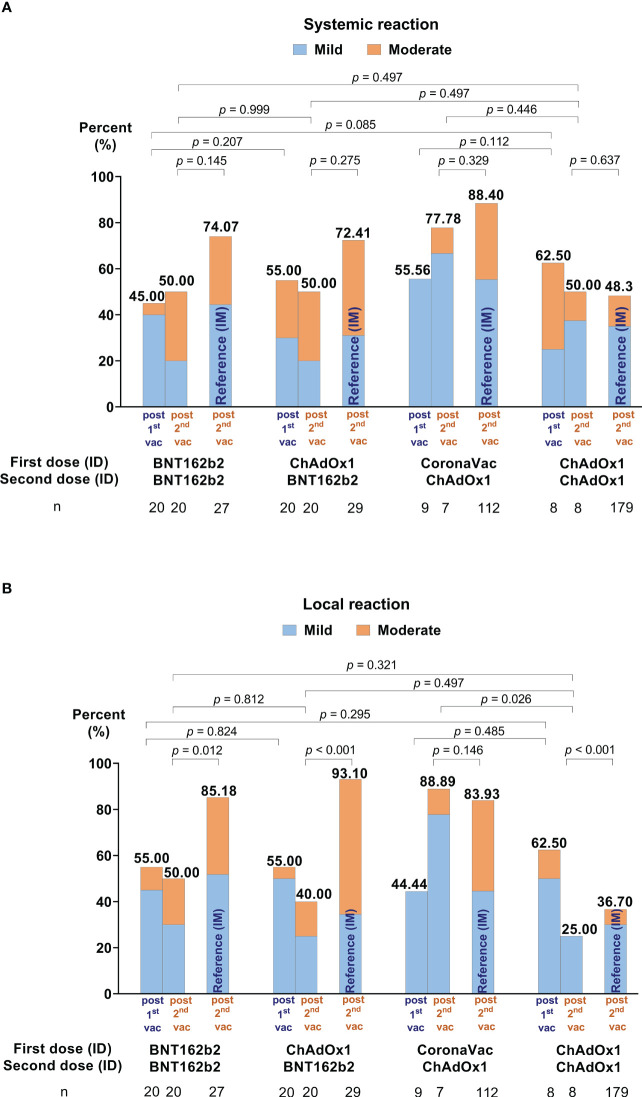
Self-reported adverse events (AEs) in days 0-7 following the first and second ID doses. **(A)** Illustrates the systemic reactions and **(B)** local effects. AEs of conventional intramuscular (IM) administration for each vaccine regimen are included as references from our previous study (15).

Many reported AEs were mild, some moderate, but none severe. All AEs fully resolved before the end of the study (see **Figures 4A, B**, **Supplementary Table 2**, and **Supplementary Figure 2**). Compared to the second IM dose, a lower proportion of systemic AEs were reported after both ID doses for each respective vaccine regimen. The only exception was homologous ChAdOx1 (Group 4), with similar systemic AEs following ID or IM administration (see **Figure 4A**). Compared to IM injection, a lower proportion of local AEs were reported for Groups 1 and 2, but not after ChAdOx1 administration in Groups 3 and 4.

## 4 Discussion

This pilot study explored the immunogenicity and reactogenicity of accelerated schedules of fractional, homologous or heterologous, ID COVID-19 immunization. Accelerated two-dose ID regimens, administered 7 days apart, as fractions of 17% (for BNT162b2) and 20% (for CoronaVac and ChAdOx1) of their standard IM dosages were immunogenic against the ancestral strain. Homologous regimens of BNT162b2-BNT162b2 and heterologous ChAdOx1-BNT162b2 induced higher humoral and cellular immune responses against the ancestral Wuhan strain than homologous ChAdOx1-ChAdOx1 and CoronaVac-ChAdOx1. However, they induced lower antibody responses than their respective conventional IM dosing (two doses, 4 weeks apart). Heterologous regimens also induced higher measurable neutralizing antibody titers than their homologous regimens. However, fractional ID dosing regimens induced poor neutralizing antibody responses against omicron subvariants, comparable to two-dose IM regimens.

Vaccine administration *via* ID routes have been used for several vaccines prior to COVID-19 (*e.g.*, rabies and hepatitis B). The dermis is rich in APCs and ID vaccination can typically achieve equivalent or superior immune responses than vaccination through IM or SC (16, 17). ID administration of low-dose regimens (10-20 µg) of mRNA-1273 were found to be safe and well tolerated, and induced robust antibody responses that were comparable to standard 100 µg mRNA-1273 IM regimens (4). Whilst immunogenic, ID administration of 20% standard IM BNT162b2 dosage as a booster induced lower antibody and cellular immune responses compared to standard IM administration in individuals previously vaccinated with CoronaVac as a primary series (2). The correlate of protection of these lower immune responses generated from ID administration is still unknown. Whether higher ID dosages (still lower than standard IM dose) will induce similar or higher immune responses than standard IM regimens should be further evaluated. We previously illustrated that ID administration of homologous or heterologous vaccine regimens of CoronaVac, ChAdOx1, and BNT162b2 as primary series four weeks apart, or boosters, were highly immunogenic and induced similar or marginally lower antibody responses than standard IM regimens (14, 18). Overall, the immunogenicity of fractional ID regimens of COVID-19 vaccines appears dependent on vaccine type, with higher responses generally found for mRNA vaccines.

Accelerated schedules provide rapid vaccine-induced immunity and have been widely used in post-exposure prophylaxis against rabies. The WHO recommends an accelerated schedule on days 0, 3, 7, and 14-28 to rapidly achieve the immune induction required to prevent rabies infection (11). Prime-boost vaccination series space dosing intervals out to ensure a long-term immune response is generated following the prime dose. ID injection has been recommended in routine practice to reduce the required volume of the administered rabies vaccine (11), an application particularly useful in resource-limited settings across Asia and Africa. Similarly, it may be applied to the COVID-19 pandemic to limit transmission within the population and thereby prevent further outbreaks. While longer intervals generally induce better immunogenicity (19), accelerated schedules may induce sufficient immunity for protection and/or priming for further boosting. We hypothesized that two ID injections at separate sites may overcome the short interval (as observed for rabies vaccinations). This study was the first to demonstrate that using accelerated schedules of two ID injections is immunogenic and may be strategically applied for rapid immune priming. Moreover, we found ID routes induced high cellular immune responses as measured by ELISpot. This again suggests that fractional, accelerated ID regimens may contain future SARS-CoV-2 outbreaks using variant-updated vaccines and appropriate vaccine regimens, particularly in instances of vaccine shortage.

Low or negligible antibody responses against omicron subvariants were observed. This was consistent with earlier findings in primary series administered as two doses IM, where boosters were required to generate robust cross immunity against omicron due to its immune evasion properties (20). Boosters are likely required after accelerated ID regimens as well to protect patients against omicron subvariants.

Additionally, as observed in other studies, homologous and heterologous fractional ID regimens were well-tolerated, with low incidences of systemic AEs and no severe local reactions (2, 15, 21). This was especially evident after the second dose (22). These findings suggest that ID administration may help reduce COVID-19 vaccine hesitancy associated with reactogenicity and safety.

Our study has some limitations. First, this is a pilot study and our sample size is small. However, we could still observe significant differences between the four vaccine groups with reference to their IM regimens. Second, we were not able to compare our data with an IM primary series within the same cohort. We minimized the variability of our findings somewhat by using reference data from the same setting, and testing methods as the previous cohort. Third, we did not have a reference IM group for ELISpot analysis. Therefore, we were unable to compare cellular immune responses between IM and ID administration directly. Fourth, there are no accelerated IM schedules with similar vaccination regimens 7 days apart for comparison. Lastly, our data may not be generalizable to other populations (*e.g.*, those > 60 years) and/or other COVID-19 vaccines.

To conclude, we found that accelerated fractional COVID-19 vaccines administered ID are immunogenic against the ancestral strain but insufficient for omicron subvariants. Our regimens were able to prime immunity, as demonstrated by humoral and cellular immune responses. With the appropriate vaccine, such strategies may be useful for containing future outbreaks, particularly in cases of vaccine shortages. Further studies on accelerated schedules warrants research on booster dose responses.

## Data availability statement

The original contributions presented in the study are included in the article/**Supplementary Material**. Further inquiries can be directed to the corresponding author.

## Ethics statement

The studies involving human participants were reviewed and approved by The Human Research Protection Unit, Faculty of Medicine Siriraj Hospital, Mahidol University (COA: MU-MOU 704/2021). The patients/participants provided their written informed consent to participate in this study.

## Author contributions

SN and KC conceptualized the study; SN, SA, PW, KS, and KC devised the methodology; SN and KC acquired funding; SN, LJ, ZT, PL, and KC carried out the formal analysis of its findings; SN, SA, PW, KS, and KC conducted the study’s clinical investigation; SN and LJ curated the data; KKC, ZT, and KC wrote and prepared the original draft; and all authors assisted with its review and editing. All authors contributed to the article and approved the submitted version.
