# Supplementary material for: Immunogenicity and reactogenicity of accelerated regimens of fractional intradermal COVID-19 vaccinations
Source: Front Immunol. 2023 Jan 17;13:1080791. doi: 10.3389/fimmu.2022.1080791 (PMC9886662; doi:10.3389/fimmu.2022.1080791)
Supplement: Supplementary file 1 [file DataSheet_1.docx]

Supplementary Material

# Supplementary Figures and Tables

## Supplementary Figures


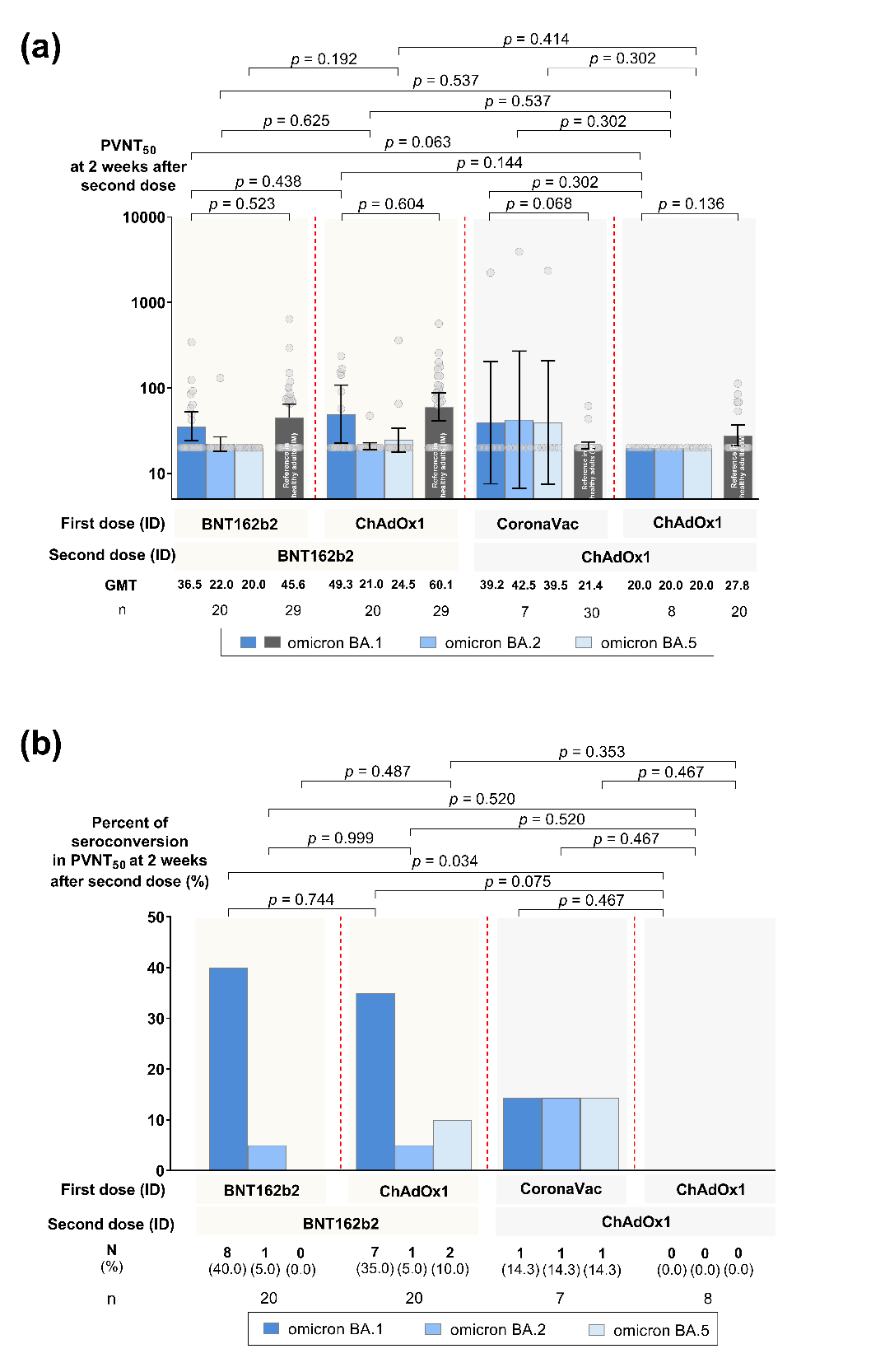


**Supplementary Figure 1.** SARS-CoV-2 humoral immune responses against the ancestral Wuhan strain following accelerated regimens of fractional ID administration. **(A)** PVNT_50_ 2 weeks after second accelerated intradermal vaccine dose. **(B)** Percent seroconversion in PVNT_50_ 2 weeks after second dose. Error bars represent geometric means (GMs) and 95% confidence intervals (CI).


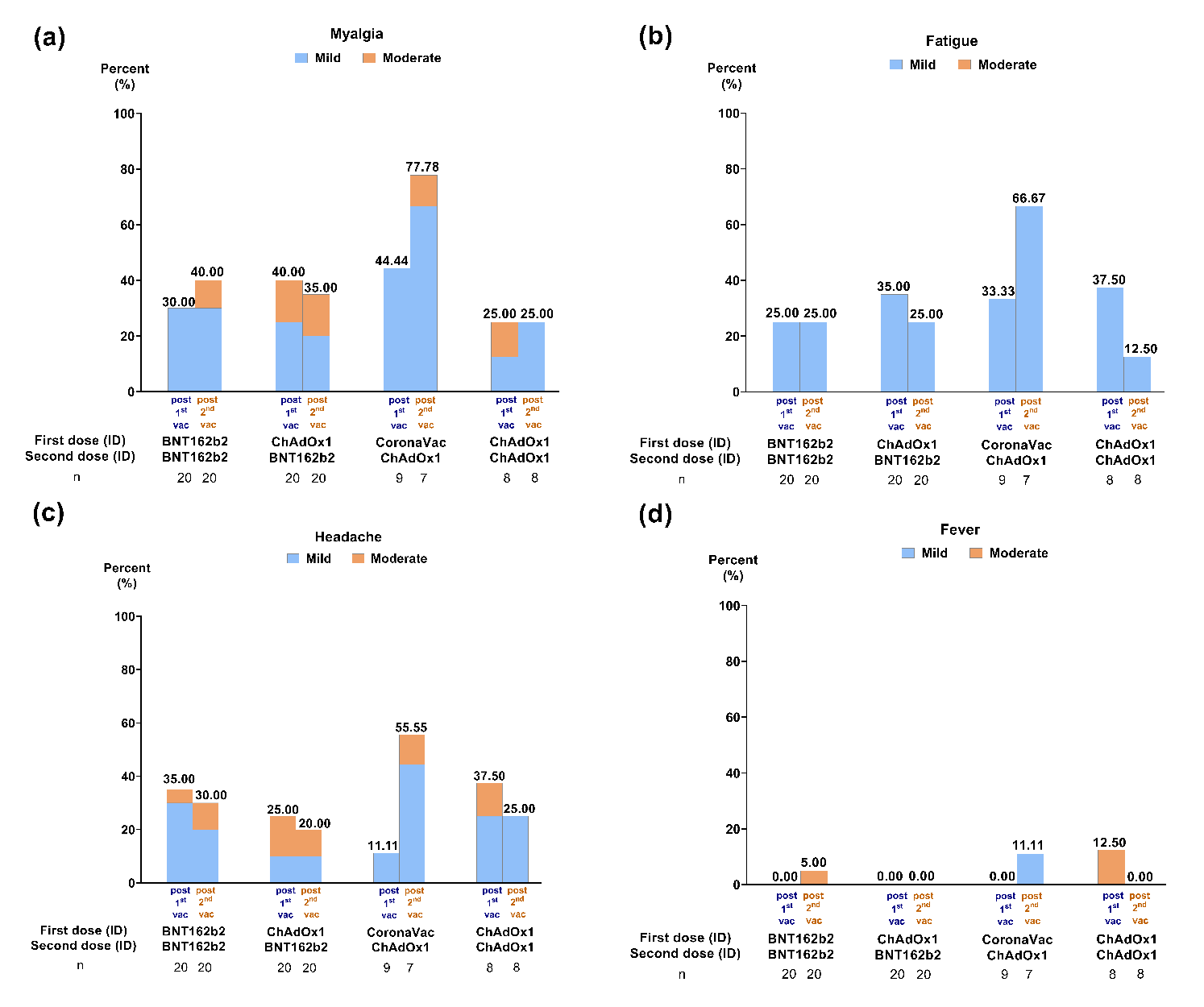
**Supplementary Figure 2.** Self-reported adverse events (AEs) in days 0-7 following the first and second ID doses.

## Supplementary Tables

Supplementary Table 1. Immunological responses following intradermal COVID-19 vaccine regimens after the first and second dose.

| **First dose (ID)**  **-**  **Second dose (ID)** | **Types of vaccines** | | | | | **p-value** |
| --- | --- | --- | --- | --- | --- | --- |
|  | **Total** | **BNT162b2**  **-**  **BNT162b2** | **ChAdOx1**  **-**  **BNT162b2** | **CoronaVac**  **- ChAdOx1** | **ChAdOx1**  **-**  **ChAdOx1** |  |
| **Anti-RBD IgG (BAU/mL)** | | | | | | |
|  | **n = 57** | **n = 20** | **n = 20** | **n = 9** | **n = 8** | **p-value** |
| GMC  at baseline (95% CI) | 0.16  (0.10, 0.25) | 0.11  (0.04, 0.27) | 0.15  (0.06, 0.38) | 0.20  (0.06, 0.61) | 0.34  (0.12, 0.98) | 0.505 |
| GMC 1 week after first dose (95% CI) | 0.18  (0.11, 0.29) | 0.15  (0.05, 0.31) | 0.15  (0.06, 0.38) | 0.24  (0.10, 0.63) | 0.44  (0.16, 1.25) | 0.340 |
|  | **n = 55** | **n = 20** | **n = 20** | **n = 7** | **n = 8** | **p-value** |
| GMC 2 weeks after second dose (95% CI) | 323.99  (241.69, 434.31) | 414.84  (316.96, 542.96) | 597.29  (411.37, 867.25) | 73.51  (44.09,  122.57) | 138.56  (43.59,  440.46) | <0.001* |
|  | **n = 30** | **n=16** | **n=14** | **n=0** | **n=0** | **p-value** |
| GMC 12 weeks after second dose (95% CI) | 144.00  (111.37, 186.19) | 133.09  (83.29, 212.66) | 157.57  (126.26, 196.66) | - | - | 0.512 |
|  | **n = 55** | **n = 20** | **n = 20** | **n = 7** | **n = 8** | **p-value** |
| **GMR between 2 weeks after second dose and baseline**  **(95% CI)** | **2,070.21**  **(1149.93,**  **3726.98)** | **3,789.19**  **(1483.59, 9677.87)** | **3,919.96**  **(1365.24, 11255.22)** | **378.73**  **(92.97, 1542.72)** | **409.25**  **(136.13,**  **1230.33)** | **0.005*** |
| **50% Pseudovirus neutralization titers (PVNT_50_) at 2 weeks after second dose** | | | | | | |
|  | **n = 55** | **n = 20** | **n = 20** | **n = 7** | **n = 8** | **p-value** |
| GMT against Wuhan strain at 2 weeks after second dose  (95% CI) | 114.84  (77.43,  170.32) | 78.12  (44.96,  135.74) | 254.20  (134.28,  447.75) | 127.22  (19.17,  844.42) | 41.31  (16.18,  105.52) | 0.010* |
| PVNT_50_ against Wuhan strain ≥ 1:40, n (%) | 40  (72.73) | 15  (75.00) | 18  (90.00) | 4  (57.14) | 3  (37.50) | 0.024 |
| GMT against omicron BA.1 strain at 2 weeks after second dose (95% CI) | 37.29  (26.29,  52.90) | 36.54  (24.12,  52.35) | 49.34  (22.50,  108.19) | 39.22  (7.55,  203.79) | 20.00  (20.00,  20.00) | 0.304 |
| PVNT_50_ against omicron BA.1 strain ≥ 1:40, n (%) | 16  (34.04) | 8  (40.00) | 7  (35.00) | 1  (14.29) | 0  (0.00) | 0.134 |
| GMT against omicron BA.2 strain at 2 weeks after second dose (95% CI) | 23.14  (18.85,  28.39) | 21.97  (18.06,  26.73) | 20.88  (19.08,  22.84) | 42.52  (6.72,  269.12) | 20.00  (20.00,  20.00) | 0.151 |
| PVNT_50_ against omicron BA.2 strain ≥ 1.40, n (%) | 3  (6.38) | 1  (5.00) | 1  (5.00) | 1  (14.29) | 0  (0.00) | 0.588 |
| GMT against omicron BA.5 strain at 2 weeks after second dose (95% CI) | 23.49  (19.13,  28.83) | 20.00  (20.00,  20.00) | 24.52  (17.79,  33.80) | 39.52  (7.47,  209.17) | 20.00  (20.00,  20.00) | 0.204 |
| PVNT_50_ against omicron BA.5 strain ≥ 1.40, n (%) | 3  (5.45) | 0  (0.00) | 2  (10.00) | 1  (14.29) | 0  (0.00) | 0.366 |
| **GMR between PVNT_50_ against Wuhan and omicron BA.1 strain at 2 weeks after second dose (95% CI)** | **3.08**  **(2.00,**  **4.74)** | **2.20**  **(1.26,**  **3.83)** | **4.97**  **(1.92,**  **12.85)** | **3.24**  **(0.76,**  **13.86)** | **2.07**  **(0.81,**  **5.28)** | **0.367** |
| **GMR between PVNT_50_ against Wuhan and omicron BA.2 strain at 2 weeks after second dose (95% CI)** | **4.96**  **(3.46,**  **7.12)** | **3.56**  **(2.28,**  **5.54)** | **11.74**  **(6.58,**  **20.97)** | **2.99**  **(0.66,**  **13.63)** | **2.07**  **(0.81,**  **5.08)** | **0.002*** |
| **GMR between PVNT_50_ against Wuhan and omicron BA.5 strain at 2 weeks after second dose (95% CI)** | **4.89**  **(3.39,**  **7.05)** | **3.91**  **(2.25,**  **6.79)** | **10.00**  **(5.55,**  **18.03)** | **3.22**  **(0.75,**  **13.83)** | **2.07**  **(0.81,**  **5.28)** | **0.014*** |
| **GMR between PVNT_50_ against omicron BA.1 and BA.2 strain at 2 weeks after second dose (95% CI)** | **1.61**  **(1.19,**  **2.18)** | **1.62**  **(1.20,**  **2.19)** | **2.36**  **(1.09,**  **5.13)** | **0.92**  **(0.76,**  **1.12)** | **1.00**  **(1.00,**  **1.00)** | **0.132** |
| **GMR between PVNT_50_ against omicron BA.1 and BA.5 strain at 2 weeks after second dose (95% CI)** | **1.59**  **(1.17,**  **2.16)** | **1.78**  **(1.21,**  **2.62)** | **2.01**  **(0.92,**  **4.39)** | **0.99**  **(0.97,**  **1.01)** | **1.00**  **(1.00,**  **1.00)** | **0.320** |
| **GMR between PVNT_50_ against omicron BA.2 and BA.5 strain at 2 weeks after second dose (95% CI)** | **0.99**  **(0.88,**  **1.10)** | **1.10**  **(0.90,**  **1.34)** | **0.85**  **(0.67,**  **1.08)** | **1.08**  **(0.90,**  **1.29)** | **1.00**  **(1.00,**  **1.00)** | **0.247** |
| **ELISpot responses (SFU/10^6^ cells)** | | | | | | |
|  | **n = 57** | **n = 20** | **n = 20** | **n = 9** | **n = 8** | **p-value** |
| ELISpot-S  GM at baseline (95% CI) | 1.74  (1.43, 2.11) | 1.47  (1.12, 1.94) | 2.55  (1.75, 3.71) | 1.20  (0.84, 1.70) | 1.54  (0.77, 3.07) | 0.028* |
| ELISpot-NMO  GM at baseline (95% CI) | 3.20  (2.50, 4.09) | 3.07  (2.05, 4.60) | 2.52  (1.87, 3.93) | 4.03  (1.24, 13.04) | 5.00  (2.63, 9.54) | 0.290 |
|  | **n = 55** | **n = 20** | **n = 20** | **n = 7** | **n = 8** | **p-value** |
| ELISpot-S  GM at 2 weeks after second dose (95% CI) | 305.85  (231.49,  404.08) | 373.80  (246.12,  567.72) | 441.34  (271.10,  718.47) | 85.88  (42.22,  174.70) | 224.99  (136.63,  370.50) | 0.002* |
| ELISpot-NMO  GM at 2 weeks after second dose (95% CI) | 4.36  (3.38, 5.61) | 4.04  (3.15, 5.18) | 2.93  (2.21, 3.90) | 11.40  (2.89, 44.98) | 6.12  (2.14, 17.52) | 0.004* |
|  | **n = 55** | **n = 20** | **n = 20** | **n = 7** | **n = 8** | **p-value** |
| ELISpot-S  GMR at 2 weeks after second dose and baseline (95%CI) | 172.37  (127.21,  233.56) | 254.05  (144.57, 459.15) | 172.98  (113.99, 262.49) | 68.16  (32.87, 141.35) | 145.89  (54.41, 391.14) | 0.057 |
| ELISpot-NMO  GMR at 2 weeks after second dose and baseline (95%CI) | 1.30  (1.01, 1.68) | 1.32  (0.91, 1.90) | 1.16  (0.81, 1.68) | 1.90  (0.62, 5.87) | 1.22  (0.38, 3.93) | 0.229 |

One-way ANOVA with parametric assumptions satisfied was determined using p-value. Titer values reported as below the lower limit of detection (LLOD = 1:40) were replaced with 20. p ≤ 0.05. Abbreviations: BAU/mL: binding antibody unit/mL; GMC: geometric mean concentration; GMT: geometric mean titer; GM: geometric mean; GMR: geometric mean ratio; SFU/10^6^ cells: spot forming unit per million cells.

Supplementary Table 2. Adverse events of accelerated intradermal COVID-19 vaccination.

|  | **Types of vaccines** | | | | | |
| --- | --- | --- | --- | --- | --- | --- |
| **First dose**  **-**  **Second dose** | **Total** | **BNT162b2**  **-**  **BNT162b2** | **ChAdOx1**  **-**  **BNT162b2** | **CoronaVac**  **- ChAdOx1** | **ChAdOx1**  **-**  **ChAdOx1** | **p-value** |
| **Number of participants** | **n = 57** | **n = 20** | **n = 20** | **n = 9** | **n = 8** |  |
| **After the first dose** | | | | | | |
| **Injection site reaction, n (%)**  Mild, n (%)  Moderate, n (%) | 32 (56.14)  28 (49.12)  4 (7.02) | 11 (55.00)  9 (45.00)  2 (10.00) | 12 (55.00)  11 (50.00)  1 (5.00) | 4 (44.44)  4 (44.44)  0 (0.00) | 5 (62.50)  4 (50.00)  1 (12.50) | 0.942 |
| **Systemic reaction, n (%)**  Mild, n (%)  Moderate, n (%) | 30 (52.63)  21 (36.84)  9 (15.79) | 9 (45.00)  8 (40.00)  1 (5.00) | 11 (55.00)  6 (30.00)  5 (25.00) | 5 (55.56)  5 (55.56)  0 (0.00) | 5 (62.50)  2 (25.00)  3 (37.50) | 0.219 |
| **Myalgia, n (%)**  Mild, n (%)  Moderate, n (%) | 20 (35.09)  16 (28.07)  4 (7.02) | 6 (30.00)  6 (30.00)  0 (0.00) | 8 (40.00)  5 (25.00)  3 (15.00) | 4 (44.44)  4 (44.44)  0 (0.00) | 2 (25.00)  1 (12.50)  1 (12.50) | 0.397 |
| **Fatigue, n (%)**  Mild, n (%)  Moderate, n (%) | 18 (31.58)  18 (31.58)  0 (0.00) | 5 (25.00)  5 (25.00)  0 (0.00) | 7 (35.00)  7 (35.00)  0 (0.00) | 3 (33.33)  3 (33.33)  0 (0.00) | 3 (37.50)  3 (37.50)  0 (0.00) | 0.885 |
| **Headache, n (%)**  Mild, n (%)  Moderate, n (%) | 16 (28.07)  11 (19.30)  5 (8.77) | 7 (35.00)  6 (30.00)  1 (5.00) | 5 (25.00)  2 (10.00)  3 (15.00) | 1 (11.11)  1 (11.11)  0 (0.00) | 3 (37.50)  2 (25.00)  1 (12.50) | 0.509 |
| **Fever, n (%)**  Mild, n (%)  Moderate, n (%) | 1 (1.75)  0 (0.00)  1 (1.75) | 0 (0.00)  0 (0.00)  0 (0.00) | 0 (0.00)  0 (0.00)  0 (0.00) | 0 (0.00)  0 (0.00)  0 (0.00) | 1 (12.50)  0 (0.00)  1 (12.50) | 0.101 |
| **Diarrhea, n (%)**  Mild, n (%)  Moderate, n (%) | 1 (1.75)  1 (1.75)  0 (0.00) | 0 (0.00)  0 (0.00)  0 (0.00) | 0 (0.00)  0 (0.00)  0 (0.00) | 1 (11.11)  1 (11.11)  0 (0.00) | 0 (0.00)  0 (0.00)  0 (0.00) | 0.143 |
| **Nausea, n (%)**  Mild, n (%)  Moderate, n (%) | 2 (3.51)  0 (0.00)  2 (3.51) | 2 (10.00)  0 (0.00)  2 (10.00) | 0 (0.00)  0 (0.00)  0 (0.00) | 0 (0.00)  0 (0.00)  0 (0.00) | 0 (0.00)  0 (0.00)  0 (0.00) | 0.280 |
| **After the second dose** | | | | | | |
| **Injection site reaction, n (%)**  Mild, n (%)  Moderate, n (%) | 28 (49.12)  20 (35.09)  8 (14.04) | 10 (50.00)  6 (30.00)  4 (20.00) | 8 (40.00)  5 (25.00)  3 (15.00) | 8 (88.89)  7 (77.78)  1 (11.11) | 2 (25.00)  2 (25.00)  0 (0.00) | 0.077 |
| **Systemic reaction, n (%)**  Mild, n (%)  Moderate, n (%) | 31 (54.39)  17 (29.82)  14 (24.56) | 10 (50.00)  4 (20.00)  6 (30.00) | 10 (50.00)  4 (20.00)  6 (30.00) | 7 (77.78)  6 (66.67)  1 (11.11) | 4 (50.00)  3 (37.50)  1 (12.50) | 0.207 |
| **Myalgia, n (%)**  Mild, n (%)  Moderate, n (%) | 24 (42.11)  18 (31.58)  6 (10.34) | 8 (40.00)  6 (30.00)  2 (10.00) | 7 (35.00)  4 (20.00)  3 (15.00) | 7 (77.78)  6 (66.67)  1 (11.11) | 2 (25.00)  2 (25.00)  0 (0.00) | 0.218 |
| **Fatigue, n (%)**  Mild, n (%)  Moderate, n (%) | 17 (29.82)  17 (29.82)  0 (0.00) | 5 (25.00)  5 (25.00)  0 (0.00) | 5 (25.00)  5 (25.00)  0 (0.00) | 6 (66.67)  6 (66.67)  0 (0.00) | 1 (12.50)  1 (12.50)  0 (0.00) | 0.059 |
| **Headache, n (%)**  Mild, n (%)  Moderate, n (%) | 17 (29.82)  12 (21.05)  5 (8.77) | 6 (30.00)  4 (20.00)  2 (10.00) | 4 (20.00)  2 (10.00)  2 (10.00) | 5 (55.55)  4 (44.44)  1 (11.11) | 2 (25.00)  2 (25.00)  0 (0.00) | 0.476 |
| **Fever, n (%)**  Mild, n (%)  Moderate, n (%) | 2 (3.51)  1 (1.75)  1 (1.75) | 1 (5.00)  0 (0.00)  1 (5.00) | 0 (0.00)  0 (0.00)  0 (0.00) | 1 (11.11)  1 (11.11)  0 (0.00) | 0 (0.00)  0 (0.00)  0 (0.00) | 0.296 |
| **Diarrhea, n (%)**  Mild, n (%)  Moderate, n (%) | 1 (1.75)  1 (1.75)  0 (0.00) | 0 (0.00)  0 (0.00)  0 (0.00) | 0 (0.00)  0 (0.00)  0 (0.00) | 1 (11.11)  1 (11.11)  0 (0.00) | 0 (0.00)  0 (0.00)  0 (0.00) | 0.143 |
| **Nausea, n (%)**  Mild, n (%)  Moderate, n (%) | 2 (3.51)  2 (3.51)  0 (0.00) | 0 (0.00)  0 (0.00)  0 (0.00) | 0 (0.00)  0 (0.00)  0 (0.00) | 1 (11.11)  1 (11.11)  0 (0.00) | 1 (12.50)  1 (12.50)  0 (0.00) | 0.179 |

Chi-squared tests were used to determined p-value.
